# Efficacy of an Internet-Delivered Intervention for Improving Insomnia Severity and Functioning in Veterans: Randomized Controlled Trial

**DOI:** 10.2196/50516

**Published:** 2023-11-24

**Authors:** Sarra Nazem, Sean M Barnes, Jeri E Forster, Trisha A Hostetter, Lindsey L Monteith, Emily B Kramer, Laurel A Gaeddert, Lisa A Brenner

**Affiliations:** 1 Dissemination & Training Division National Center for Posttraumatic Stress Disorder Menlo Park, CA United States; 2 Veterans Affairs Rocky Mountain Mental Illness Research, Education and Clinical Center Aurora, CO United States; 3 Department of Psychiatry University of Colorado Anschutz Medical Campus Aurora, CO United States; 4 Department of Physical Medicine and Rehabilitation University of Colorado Anschutz Medical Campus Aurora, CO United States; 5 Department of Neurology University of Colorado Anschutz Medical Campus Aurora, CO United States

**Keywords:** cognitive behavioral therapy, insomnia, internet intervention, online intervention, randomized controlled trial, RCT, RCTs, sleep, treatment, veteran, veterans, veterans’ health

## Abstract

**Background:**

Despite a growing evidence base that internet-delivered cognitive behavioral therapy for insomnia (iCBT-I) is associated with decreased insomnia severity, its efficacy has been minimally examined in veterans.

**Objective:**

The objective of this study was to evaluate the efficacy of an unguided iCBT-I (Sleep Healthy Using the Internet [SHUTi]) among veterans.

**Methods:**

We conducted a single-blind, randomized controlled trial in Operation Enduring Freedom, Operation Iraqi Freedom, and Operation New Dawn veterans eligible for Veterans Health Administration care. Participants were randomly assigned (1:1) to receive SHUTi (a self-guided and interactive program) or an Insomnia Education Website (IEW) that provided nontailored and fixed insomnia information. Web-based assessments were administered at baseline, postintervention, 6 months postintervention, and 1 year postintervention. The primary outcome was self-reported insomnia severity (Insomnia Severity Index [ISI]). Secondary outcomes were self-reported mental and physical health functioning (Veterans RAND 36-item Health Survey). Exploratory outcomes comprised sleep diary parameters.

**Results:**

Of the 231 randomized participants (mean age 39.3, SD 7.8 years; 170/231, 73.5% male sex; 26/231, 11.3% Black; 172/231, 74.5% White; 10/231, 4.3% multiracial; and 17/231, 7.4% other; 36/231, 15.6% Hispanic) randomized between April 2018 and January 2019, a total of 116 (50.2%) were randomly assigned to SHUTi and 115 (49.8%) to the IEW. In intent-to-treat analyses, SHUTi participants experienced significantly larger ISI decreases compared with IEW participants at all time points (generalized η^2^ values of 0.13, 0.12, and 0.10, respectively; all *P*<.0001). These corresponded to estimated larger differences in changes of –3.47 (95% CI –4.78 to –2.16), –3.80 (95% CI –5.34 to –2.27), and –3.42 (95% CI –4.97 to 1.88) points on the ISI for the SHUTi group. SHUTi participants experienced significant improvements in physical (6-month generalized η^2^=0.04; *P*=.004) and mental health functioning (6-month and 1-year generalized η^2^=0.04; *P*=.009 and *P*=.005, respectively). Significant sleep parameter improvements were noted for SHUTi (all *P*<.05), though the pattern and magnitude of these reductions varied by parameter. No adverse events were reported.

**Conclusions:**

Self-administered iCBT-I was associated with immediate and long-term improvements in insomnia severity. Findings suggest that leveraging technology to meet insomnia treatment demands among veterans may be a promising approach.

**Trial Registration:**

ClinicalTrials.gov NCT03366870; https://clinicaltrials.gov/ct2/show/NCT03366870

## Introduction

Insomnia is a public health and clinical problem affecting US veterans. In a nationally representative sample of US veterans, 35% endorsed symptoms consistent with clinical or subthreshold insomnia [1]. This is especially concerning given that insomnia precedes and exacerbates functionally impairing mental health concerns and is associated with suicide risk [2-7]. Chronic insomnia is also linked to an increased risk of chronic diseases (eg, diabetes, cardiovascular diseases, and cancer) [8,9]. To improve veterans’ mental and physical health, it is imperative that veterans receive evidence-based psychotherapies (EBPs) that reduce insomnia.

Cognitive behavioral therapy for insomnia (CBT-I) is one of the most efficacious treatments for insomnia [10-12]. Long-term effects surpass those of sleep medications, with the added benefit of being associated with few adverse side effects [13,14]. Despite successful Veterans Health Administration (VHA) efforts that have improved access to CBT-I, the resources necessary to implement provider-delivered CBT-I outstrip treatment demands [15-19]. Self-guided, internet-delivered cognitive behavioral therapy (iCBT) offers a potentially efficacious means of delivering insomnia treatments by providing individuals with the opportunity to engage in an EBP on the web. Veterans have an interest in digital health technologies, including internet-delivered CBT-I (iCBT-I), and leveraging technology is critical to meeting treatment demand and patient preferences [20-24].

From both policy and clinical perspectives, iCBTs have garnered attention due to their potential to overcome barriers to treatment (eg, cost, scheduling and travel demands, stigma, and lack of trained EBP clinicians) [25,26]. Recent meta-analyses found that iCBT-I improved insomnia severity, sleep parameters, and subjective sleep quality, with effect sizes comparable to those found in face-to-face (F2F) CBT-I (ie, Hedges *g* ranging from 0.21 to 1.09) [27,28]. iCBT-I programs that included more CBT-I elements, had longer intervention periods, and used highly interactive program designs optimized outcomes and facilitated better treatment engagement and adherence [29].

Despite veterans’ interest in digital health technologies and a growing evidence base for iCBT-I interventions, relatively few studies have examined iCBT-I interventions in veteran populations. Initial, formative studies examined the use of the patient mobile app, CBT-I Coach, which was developed and designed to help facilitate patients’ completion of CBT-I tasks while receiving F2F CBT-I. A pilot randomized controlled trial (RCT; n=18) demonstrated the feasibility and acceptability of integrating CBT-I Coach with individual F2F CBT-I [23]. A total of 2 additional small studies (n<35) found that self-administered use of CBT-I Coach paired with supplemental app-delivered worksheets and a self-management guide was associated with a reduction of insomnia symptoms in veterans reporting chronic insomnia [30,31]. Researchers have also demonstrated the feasibility and potential efficacy of stand-alone, self-guided iCBT-I interventions in 3 studies. First, in an uncontrolled pre-post intervention study of a 6-session self-administered iCBT-I, veterans seeking care in a Department of Veterans Affairs (VA) substance use disorder outpatient clinic achieved clinically significant improvements in insomnia severity following the iCBT-I intervention [32]. In a pilot RCT, veterans with probable insomnia disorder (n=50) were randomized to Insomnia Coach, a self-management CBT-I app, or a waitlist control. Veterans found Insomnia Coach to be feasible and acceptable, with preliminary efficacy data suggesting that app use was associated with clinically significant improvement in insomnia severity [33]. Finally, using a cohort trial design, Hermes and colleagues [34] examined the feasibility of implementing Sleep Healthy Using the Internet (SHUTi) in VA primary care clinics. Although the primary aim of this study was to examine differences in implementation outcomes based on low-intensity implementation strategy modifications, improved clinical outcomes were observed, with 20% of veterans achieving clinically meaningful change in their insomnia symptoms.

Previous studies examining iCBT-I interventions, including apps, in veteran populations have established the acceptability and feasibility of digital health interventions, with preliminary evidence suggesting that iCBT-I interventions are associated with improved insomnia outcomes as observed in nonveteran populations. Most of these studies, however, were not powered for efficacy outcomes, and no RCT has compared an evidence-based iCBT-I with a nonwaitlist control in a veteran population. Further, no study has conducted long-term follow-up beyond 12 weeks, and most studies have enrolled veterans without a confirmed insomnia diagnosis. To strengthen the evidence base, this study was designed to evaluate the efficacy of an evidence-based iCBT-I intervention, SHUTi, to decrease insomnia severity among Operation Enduring Freedom (OEF), Operation Iraqi Freedom (OIF), and Operation New Dawn (OND) veterans. Although SHUTi is one of the most empirically supported iCBT-I programs in nonveteran populations [35-44], to our knowledge, no RCT has been conducted to examine the efficacy of SHUTi in a veteran sample. We hypothesized that SHUTi participants would show greater improvements in insomnia severity and mental and physical health functioning across all time points, compared with participants randomized to an Insomnia Education Website (IEW) control. We also evaluated whether SHUTi was associated with improvements across sleep diary parameters (exploratory objective).

## Methods

### Ethical Considerations

The study design and protocol (Multimedia Appendix 1 [2,15,25,29,40-75]) were approved by the following supporting agencies: Colorado Multiple Institutional Review Board (COMIRB 17-0920), Rocky Mountain Regional Veterans Affairs Medical Center Research and Development Committee, and the US Army Medical Research and Development Command Human Research Protection Office (HRPO Log Number A-19051.2; ORHO Log Number E03947.2). Additional information on the informed consent process, data privacy and security measures, and compensation are provided below and in Multimedia Appendix 1.

### Trial Design

This is a single-blind (participants blinded to treatment allocation) 2-group (SHUTi vs IEW) longitudinal (4 time points) RCT. Assessments were administered at baseline (T1), 9 weeks postintervention (T2), 6 months postintervention (T3), and 1 year postintervention (T4). Data were collected on the web, and participants provided informed consent before study enrollment. The RCT was registered with ClinicalTrials.gov (NCT03366870).

### Randomization

Due to the empirical association between insomnia and suicide risk, randomization was stratified by self-reported suicide attempt history (“yes” or “no”). A statistically generated allocation sequence, using random blocks of 4 and 6, stratified by suicide attempt history, was conducted using SAS PROC PLAN (SAS Institute). Randomization tables were generated by the trial’s statistician and programmed to automatically assign a participant to the next allocation immediately after T1 completion.

### Participants

OEF, OIF, and OND veterans aged between 18 and 55 years were eligible to participate if they met the following criteria: current *Diagnostic and Statistical Manual of Mental Disorders, Fifth Edition* (DSM-5) [45] insomnia diagnosis; reliable access to the internet; eligibility to receive VHA care; English-speaking; and ability to provide informed consent. Exclusion criteria included the following: being currently enrolled in another intervention research study, the presence of another untreated sleep disorder, currently receiving psychological treatment for insomnia (excluding sleep medications), a change in schedule or dosage of sleep medications in the past 3 months, and the presence of a condition contraindicated with CBT-I. Examples of conditions contraindicated with CBT-I included untreated seizures or seizure disorder, pregnancy or a plan to become pregnant, irregular shift work, and significant cognitive impairment. Veterans with comorbid psychiatric presentations were eligible to participate in the RCT unless they had used any nonalcohol substances (excluding cannabis) more than once within the past 3 months; endorsed a problematic pattern of alcohol use associated with significant impairment or distress (past 3 months); or had ever been diagnosed with bipolar I disorder, schizophrenia, schizoaffective, or a psychotic disorder. Those with comorbid medical conditions were included unless conditions were deemed active, unstable, or degenerative in a manner expected to influence sleep. Research staff received extensive training on conducting semistructured interviews focused on clinical sleep history to inform differential diagnoses (eg, untreated sleep apnea, periodic limb movement disorder, parasomnia, and circadian rhythm disorder) and structured clinical interviews for DSM-5 Research Version [76] modules (eg, insomnia disorder and alcohol use disorder) to determine RCT eligibility; cases were regularly reviewed by the team and principal investigator to ensure accuracy.

Total compensation for completion of all study assessments was US $208, and the participant payment for each study assessment was as follows: US $1 for each preintervention sleep diary (up to US $14 maximum), US $50 for T1 and T2 assessments, US $1 for each postintervention diary (up to US $14 maximum), and US $40 for T3 and T4 assessments.

### Outcomes

The primary outcome measure was the Insomnia Severity Index (ISI) [77], one of the most empirically examined and clinically used self-report scales assessing insomnia symptoms. The ISI is comprised of 7 items that assess current (ie, past 2 weeks) insomnia severity. Each item is scored on a 0-4 scale, with total scores ranging from 0-28; each 7-point increment is associated with increasing levels of insomnia severity. The ISI has been shown to have adequate test-retest reliability over 3 months and concurrent validity with sleep diaries and polysomnography [46] and has been validated for web-based use [78]. In the current sample, the ISI demonstrated adequate internal consistency (Cronbach α ranged from .78 to .90 across the 4 time points).

Our secondary outcome measure was the Veterans RAND 36-item Health Survey (VR-36) [79]. The VR-36 assesses perceived general health-related quality of life; respondents indicate the impact of health on physical functioning, role limitations due to physical problems, bodily pain, general health perceptions, vitality, social functioning, role limitations due to emotional problems, and mental health. The VR-36 was formerly called the Short Form Health Survey for Veterans (Veterans SF-36) [80]. The VR-36 has been thoroughly validated and has strong psychometric properties [79,80]. Scores are used to derive 2 summary scales (ie, Physical Component Summary [PCS] and Mental Component Summary [MCS]). Scores range from 0-100, with a mean score of 50 (SD 10). Lower scores indicate poorer health-related quality of life.

### Exploratory Outcomes

Daily sleep diaries based on consensus sleep diary [81] questions were collected for 2 weeks before T1 (preintervention sleep diaries) and immediately following T2 (postintervention sleep diaries). Sleep parameters included sleep onset latency (SOL; time to fall asleep), wake after sleep onset (WASO) + early morning awakenings (EMA; combined time awake after falling asleep and before final awakening), time in bed (TIB), total sleep time (TST), and sleep efficiency (percentage of time asleep while in bed).

### Clinical Characteristics

A total of 4 psychometrically sound assessments (Multimedia Appendix 2 [47,48,82-85]) were used to characterize baseline clinical characteristics: (1) Adult Suicidal Ideation Questionnaire [47], a 25-item self-report instrument that assesses past-month suicidal ideation frequency; (2) Beck Anxiety Inventory [48], a 21-item self-report rating inventory that measures past-week subjective, somatic, and panic-related symptoms of anxiety; (3) Beck Depression Inventory II [82], a 21-item self-report rating inventory that measures past 2-week characteristic attitudes and symptoms of depression; and (4) Posttraumatic Stress Disorder Checklist for DSM-5 (PCL-5) [83], a 20-item self-report measure that assesses past-month severity of posttraumatic stress disorder symptoms.

### Interventions

#### SHUTi Program

SHUTi is a self-guided, interactive, and tailored internet-delivered program modeled on the primary tenants of F2F CBT-I [44]. Intervention content is delivered through 6 “cores” where users obtain access to a new core based on a time- and event-based schedule. The SHUTi program relies on user-entered web-based sleep diaries to track progress and tailor treatment recommendations. Each SHUTi core acts as a web-based analog for the weekly sessions typically used when delivering CBT-I in a F2F format, following the same general structure: (1) core objectives (what will be learned and why this information is important), (2) review of previous week’s homework and sleep diary data, (3) new intervention material, (4) assignment of homework (treatment strategies for the coming week), and (5) a summary of the core’s main points. Intervention content is enhanced through a variety of interactive features, including personalized goal setting, graphical feedback based on inputted symptoms, animations and illustrations to enhance comprehension, quizzes to test users’ knowledge, patient vignettes, and video-based expert explanation. The SHUTi program relies on user-entered web-based sleep diaries to track progress and tailor treatment recommendations. Automated emails are sent throughout the intervention to encourage program adherence.

#### IEW Control

The IEW used in this RCT has been previously used in SHUTi trials [37,44]. Informed by what typically constitutes “treatment as usual” for patient education websites targeting insomnia, the IEW provided static information about insomnia symptoms, causes, and strategies to improve sleep (ie, sleep hygiene, stimulus control, and behavioral recommendations). In contrast to SHUTi, IEW strategies were presented as text with no tailoring, interactivity, or feedback.

### Procedure

Veterans were screened over the phone for eligibility. Of the 844 veterans who expressed interest in the study, a total of 61 were unable to be reached for screening, and 494 were ineligible for participation. The top reasons for ineligibility were identification of another untreated sleep disorder (162/494, 32.8%); not serving in the OEF, OIF, or OND conflict (112/494, 22.7%); multiple exclusion criteria (54/494, 10.9%); and not meeting criteria for insomnia disorder (52/494, 10.5%). After screening, a total of 289 veterans were eligible for the study, and 250 were enrolled.

Most participants (234/250, 93.6%) enrolled were recruited from targeted mailings sent to veterans with diagnosed sleep problems, as identified by medical codes in the VA’s Corporate Data Warehouse. An additional 3.6% (9/250) of enrolled participants were recruited based on their expressed interest in learning more about the study. Participants were enrolled in the RCT between April 2018 and January 2019. The final T4 assessment was completed in June 2020.

After providing informed consent, participants were given instructions on how to complete a web-based, 2-week sleep diary assessment and informed that they had to complete 10 out of 14 sleep diaries to move forward in the study; a total of 6% (15/250) of enrolled veterans did not complete the sleep diary requirement. Baseline assessments (T1) were unlocked once the requirement was fulfilled. A total of 3 participants did not complete T1 assessments, resulting in a total of 231 participants (92.4%) who were randomized.

**Figure 1:**
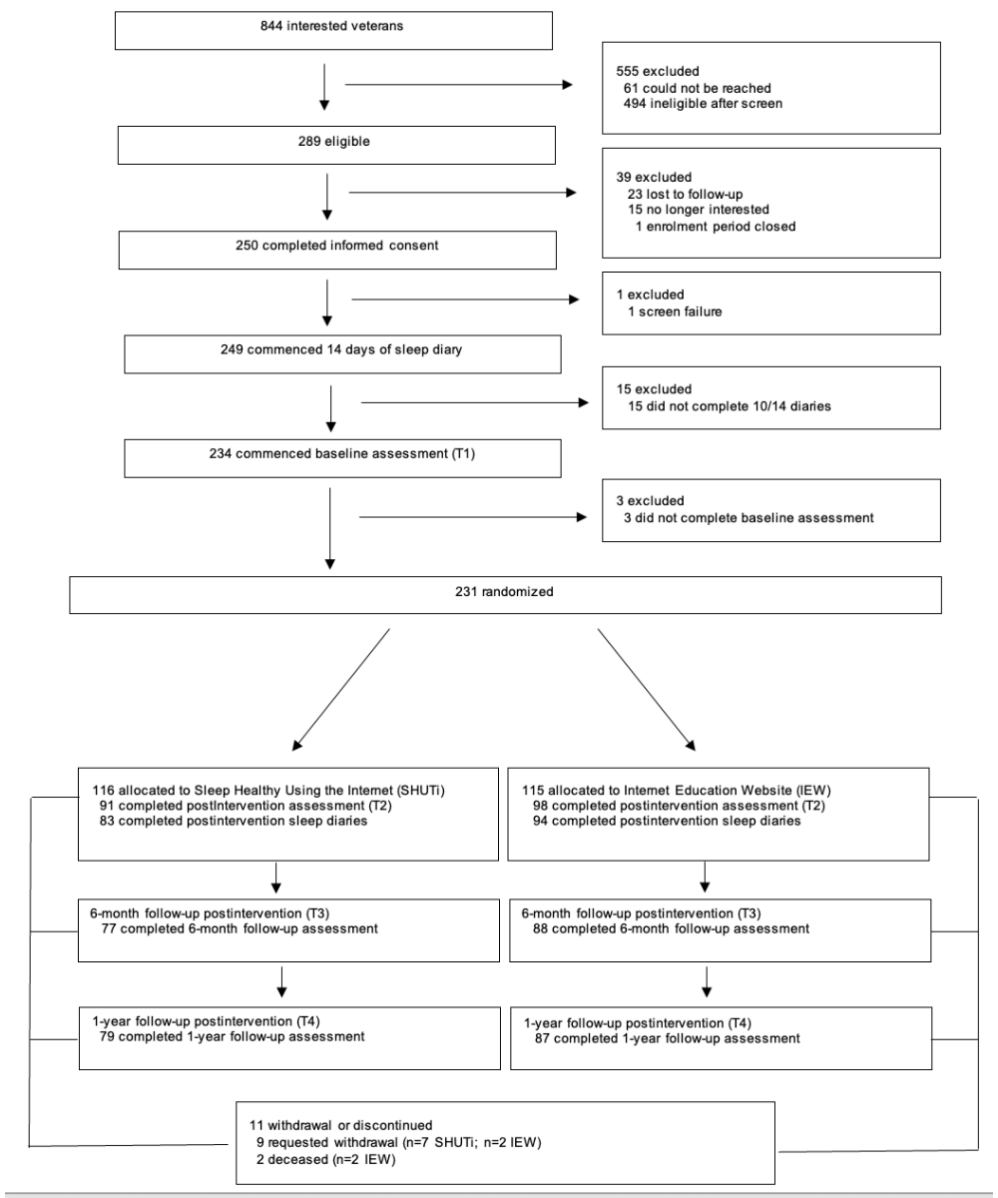
CONSORT (Consolidated Standards of Reporting Trials) diagram of study enrollment flow. IEW: Insomnia Education Website; SHUTi: Sleep Healthy Using the Internet.

A total of 84.8% (196/231) of all randomized participants completed at least 1 postintervention assessment, of which 151 participants completed all 3 assessments (77%), another 21 completed 2 assessments (10.7%), and 24 completed 1 assessment (12.2%). A total of 9 participants asked to be withdrawn from the study: 1 during the intervention, 2 at T2, 5 at T3, and 1 at T4. A total of 2 participants randomized to the IEW died during the study (deaths unrelated to research procedures); these deaths occurred between T2 and T3, and T3 and T4. Figure 1 depicts the study enrollment flow.

Participants received reminder calls from study staff to complete initial sleep diaries and the T1 assessment. During the 9-week intervention window, study staff did not initiate any contact with participants, so participants only received automated emails built into the interventions during the intervention window. Participants received reminder calls, automated emails, and reminder postcards to support postintervention (T2-T4) assessment completion.

Participants were compared across demographic variables, ISI, and VR-36 summary scale scores to examine whether there were differences between participants who did not complete a postintervention assessment (n=35) and participants who completed at least 1 postintervention assessment (n=196). The only statistically significant difference observed was that participants who did not complete a postintervention assessment were younger (mean 35.4, SD 6.4 years) than participants who completed at least 1 postintervention assessment (mean 40.0, SD 7.8 years; 2-tailed t_229_=3.33; *P*=.001).

Of the 35 participants who did not complete a postintervention assessment, a total of 20 randomized to SHUTi demonstrated significantly lower VR-36 MCS scores (mean 31.9, SD 8.2) than the 15 randomized to the IEW (mean 40.1, SD 12.6; 2-tailed t_33_=2.35; *P*=.02). No other statistically significant differences were observed between groups.

### Statistical Methods

Because data were collected on the web, steps were taken to verify data quality before conducting analyses, including examining abnormally low survey completion times and surveys with potentially biased or inconsistent responses. No inconsistent, biased, or inaccurate response patterns were identified in the data set. Missing data was minimal across measures and was handled in accordance with manual instructions. Sleep diaries were excluded from analyses if participants provided too few (less than 7) usable sleep diaries during the sleep diary assessment window (T1, n=2; and T2, n=4).

We used any pair power and Holm sequential procedure [86] to control the familywise error rate, considering all 3 primary (ISI: T2-T4) and 6 secondary outcomes (PCS: T2-T4, and MCS: T2-T4). A final sample size of at least 170 completing T2 assessments (85 per group) would provide 80% power to detect an effect size of 0.6 for each outcome. Assuming an attrition rate of 25%, we sought to randomize at least 226 participants to achieve at least 85 per group completing T2 assessments.

The analysis of covariance was used to model the change from T1 to T2, from T1 to T3, and from T1 to T4 for each outcome as a function of group, the baseline value of the respective outcome, and suicide attempt (stratification variable). Generalized η^2^ [87] is reported with all analysis of covariance results, where values of 0.01 are considered small, 0.06 as medium, and 0.14 as large. Fisher exact tests were used for post hoc analyses that examined the clinical significance of ISI changes at each postintervention assessment. All analyses were conducted using SAS (version 9.4; SAS Institute) statistical software [88].

## Results

### Overview

There were no significant differences between groups on demographic or baseline clinical characteristics (Table 1 and Multimedia Appendix 2). At T1, participants on average endorsed moderate insomnia and reported deficits in functioning, with PCS and MCS scores about 1 SD below the normative US population sample but consistent with the VHA normative sample. Table 2 contains the ISI, PCS, and MCS descriptive statistics over time.

**Table 1:** Demographic and baseline clinical characteristics.

		Sleep Healthy Using the Internet (n=116)	Insomnia Education Website (n=115)	*P* value	
Age (years), mean (SD)		39.1 (8.0)	39.5 (7.6)	.72	
Male sex, n (%)		86 (74)	86 (7)	.91	
**Gender^a^, n (%)**					.88^b^
	Man	84 (73)	86 (75)		
	Woman	30 (26)	29 (25)		
	Transwoman	1 (1)	0 (0)		
**Race^c^, n (%)**					.92
	Black or African American	13 (12)	13 (12)		
	White or Caucasian	85 (76)	87 (77)		
	Multiracial	6 (5)	4 (4)		
	Other	8 (7)	9 (8)		
Hispanic^d^, n (%)		16 (14)	20 (18)	.45	
**Relationship status, n (%)**					.74
	Married	67 (58)	67 (58)		
	Single	16 (14)	21 (18)		
	Cohabitating	11 (9)	9 (8)		
	Widowed, divorced, or separated	22 (19)	18 (16)		
**Employment, n (%)**					.96
	Full time	68 (59)	65 (57)		
	Part time	9 (8)	8 (7)		
	Unemployed and not seeking	10 (9)	10 (9)		
	Unemployed and seeking	13 (11)	12 (10)		
	Retired	16 (14)	20 (17)		
Deployed		99 (85)	106 (92)	.10	
Combat^a^		87 (76)	87 (76)	>.99	
**Era (not mutually exclusive), n (%)**					
	Post-Vietnam	10 (9)	9 (8)	.83	
	Desert Storm	34 (29)	29 (25)	.48	
	OEF^e^ or OIF^f^	113 (97)	113 (98)	>.99^b^	
	Other	1 (1)	1 (1)	>.99^b^	
History of a suicide attempt, mean (SD)		23 (20)	23 (20)	.97	
ISI^g^ total score, mean (SD)		16.9 (4.6)	16.3 (4.3)	.35	
Veterans SF-36^h^ PCS^i^, mean (SD)		41.8 (10.1)	42.9 (9.7)	.40	
Veterans SF-36 MCS^j^, mean (SD)		37.0 (12.4)	37.3 (11.6)	.86	
ASIQ^k^ total score^d^, mean (SD)		14.4 (20.4)	13.2 (18.4)	.64	
BAI^l^ total score, mean (SD)		15.6 (13.4)	15.3 (12.5)	.83	
BDI^m^ total score^n^, mean (SD)		20.8 (12.6)	19.7 (12.1)	.49	
PCL-5^o^ total sore, mean (SD)		34.6 (18.6)	33.0 (19.3)	.51	
Provisional PTSD^p^, n (%)		66 (57)	53 (46)	.10	
Probable PTSD, n (%)		61 (53)	53 (46)	.32	

^a^n=115, Sleep Healthy Using the Internet.

^b^Fisher exact test.

^c^n=112, Sleep Healthy Using the Internet; n=113, Insomnia Education Website.

^d^n=115, Sleep Healthy Using the Internet; n=114, Insomnia Education Website.

^e^OEF: Operation Enduring Freedom.

^f^OIF: Operation Iraqi Freedom.

^g^ISI: Insomnia Severity Index.

^h^Veteran SF-36: Short Form Health Survey for Veterans.

^i^PCS: Physical Component Summary.

^j^MCS: Mental Component Summary.

^k^ASIQ: Adult Suicidal Ideation Questionnaire.

^l^BAI: Beck Anxiety Inventory.

^m^BDI: Beck Depression Inventory.

^n^n=114, Insomnia Education Website.

^o^PCL-5: Posttraumatic Stress Disorder Checklist for Diagnostic and Statistical Manual of Mental Disorders, Fifth Edition

^p^PTSD: posttraumatic stress disorder.

**Table 2:** Primary and secondary outcome descriptive statistics over time.

Outcome				Time 1				Time 2							Time 3				Time 4				
				n		Frequency	n					Frequency		n			Frequency	n				Frequency	
**Sleep Healthy Using the Internet**																							
	ISI^a^, mean (SD)			116		16.9 (4.6)				91		11.6 (6.7)		77			10.5 (6.8)				79		10.7 (6.7)
	**ISI categorical, n (%)**			116						91				77							79		
		0-7			1 (1)						28 (31)					29 (38)						32 (41)	
		8-14			40 (34)						33 (36)					27 (35)						26 (33)	
		15-21			54 (47)						23 (25)					15 (19)						15 (19)	
		22-28			21 (18)						7 (8)					6 (8)						6 (8)	
	PCS^b^, mean (SD)			116		41.8 (10.1)				91		42.5 (10.5)		77			43.7 (10.6)				78		42.6 (10.2)
	MCS^c^, mean (SD)			116		37.0 (12.4)				91		42.5 (13.4)		77			43.9 (13.8)				78		43.7 (13.2)
**Insomnia Education Website**																							
	ISI, mean (SD)			115		16.3 (4.3)				98		14.9 (4.9)		88			13.9 (5.1)				87		13.8 (4.8)
	**ISI categorical, n (%)**			115						98				88							87		
		0-7			2 (2)						4 (4)					6 (7)						11 (13)	
		8-14			40 (35)						45 (46)					48 (55)						40 (46)	
		15-21			57 (50)						41 (42)					25 (28)						29 (33)	
		22-28			16 (14)						8 (8)					9 (10)						7 (8)	
	PCS, mean (SD)			115		42.9 (9.7)				97		41.8 (9.7)		88			42.3 (8.9)				87		42.0 (10.4)
	MCS, mean (SD)			115		37.3 (11.6)				97		38.7 (12.5)		88			38.6 (11.7)				87		38.2 (12.2)

^a^ISI: Insomnia Severity Index.

^b^PCS: Physical Component Summary of the Veterans RAND 36-item Health Survey.

^c^MCS: Mental Component Summary of the Veterans RAND 36-item Health Survey.

### Primary and Secondary Outcomes

SHUTi participants experienced a significantly larger T1 to T2 (–3.47, 95% CI –4.78 to –2.16 points; *P*<.0001), T1 to T3 (–3.80, 95% CI –5.34 to –2.27 points; *P*<.0001) and T1 to T4 (–3.42, 95% CI –4.97 to –1.88 points; *P*<.0001) decreases on the ISI compared with IEW participants. The magnitude of the results was maintained across all time points (generalized η^2^ values of 0.13, 0.12, and 0.10; Table 3).

SHUTi participants experienced a significantly greater T1 to T3 improvement on the VR-36 PCS (3.22, 95% CI 1.07-5.36 points; generalized η^2^=0.04; *P*=.004) compared with IEW participants (Table 3). Significance was not observed for the change from T1 to T2 (*P*=.03) or from T1 to T4 (*P*=.14). For the VR-36 MCS, SHUTi participants had a significantly greater T1 to T3 (4.33, 95% CI 1.11-7.55 points; generalized η^2^=0.03; *P*=.009) and T1 to T4 (4.53, 95% CI 1.39-7.67 points; generalized η^2^=0.04; *P*=.005) improvement, but the difference in change from T1 to T2 did not achieve statistical significance (2.58, 95% CI –0.07 to 5.24 points; *P*=.06; Table 3).

**Table 3:** Estimated difference in change between groups. All models control for the baseline value of the outcome and history of suicide attempt (“yes” or “no”). Dependent variables were calculated as the later time point minus baseline, and the difference in change was estimated as Sleep Healthy Using the Internet minus Insomnia Education Website. The italicized text indicates statistical significance based on the Holm sequential procedure, considering all 9 outcomes.

Models	Change time 1 to time 2^a,b^				Change time 1 to time 3^c^				Change time 1 to time 4^d,e^		
	Estimated difference between groups, SE	Generalized η^2^	*P* value	Estimated difference between groups, SE		Generalized η^2^	*P* value	Estimated difference between groups, SE		Generalized η^2^	*P* value
ISI^f^	–3.47 (0.66)	0.13	*<.0001*	–3.80 (0.78)		0.12	*<.0001*	–3.42 (0.78)		0.10	*<.0001*
PCS^g^	2.07 (0.96)	0.02	.03	3.22 (1.08)		0.04	*.004*	1.81 (1.22)		0.01	.14
MCS^h^	2.58 (1.35)	0.02	.06	4.33 (1.63)		0.04	*.009*	4.53 (1.59)		0.04	*.005*

^a^n=98, Insomnia Education Website (for ISI); n=91, Sleep Healthy Using the Internet (for ISI).

^b^n=97, Insomnia Education Website (for PCS and MCS); n=91, Sleep Healthy Using the Internet (for PCS and MCS).

^c^n=88, Insomnia Education Website (for all outcomes); n=77, Sleep Healthy Using the internet (for all outcomes).

^d^n=87, Insomnia Education Website (for ISI); n=79, Sleep Healthy Using the Internet (for ISI).

^e^n=87, Insomnia Education Website (for PCS and MCS); n=78, Sleep Healthy Using the Internet (for PCS and MCS).

^f^ISI: Insomnia Severity Index.

^g^PCS=Physical Component Summary of the Veterans RAND 36-item Health Survey.

^h^MCS=Mental Component Summary of the Veterans RAND 36-item Health Survey.

### Exploratory Outcomes: Sleep Diary Parameters

Results for the sleep diary parameters are displayed in Table 4. SHUTi participants experienced a significantly greater T1 to T2 decrease in SOL (*P*=.0001), WASO+EMA (*P*=.006), TIB (*P*<.0001), and TST (*P*=.04), compared with IEW participants, and a significantly greater improvement in sleep efficiency (*P*=.02). Specifically, SOL decreased by 16.9 (95% CI –25.6 to –8.3; generalized η^2^=0.04) more minutes for SHUTi participants, WASO+EMA decreased by 15.3 (95% CI –26.1 to –4.4; generalized η^2^=0.03) minutes more, TIB decreased by 48 (95% CI –65.4 to –30.0; generalized η^2^=0.12) minutes more, and TST decreased by 18.6 (95% CI –36.6 to –0.60; generalized η^2^=0.02) minutes more. Sleep efficiency improved for SHUTi participants 3.74% (95% CI 0.72%-6.75%; generalized η^2^=0.03) more than for the IEW participants.

**Table 4:** Exploratory sleep parameter models. All models control for the baseline value of the outcome and history of suicide attempt (“yes” or “no”). Dependent variables were calculated as the later time point minus baseline, and the difference in change was estimated as Sleep Healthy Using the Internet minus Insomnia Education Website. The italicized text indicates statistical significance at the *P*<.05 level.

Outcome	Change Time 1 to Time 2^a^		
	Group parameter estimate, SE	Generalized η^2^	*P* value
Sleep onset latency (minutes)	–16.95 (4.37)	0.04	*.0001*
Wake after sleep onset + early morning awakenings (minutes)	–15.26 (5.51)	0.03	*.006*
Time in bed (hours)	–0.80 (0.15)	0.12	*<.0001*
Total sleep time (hours)	–0.31 (0.15)	0.02	*.04*
Sleep efficiency (%)	3.74 (1.53)	0.03	*.02*

^a^n=89, Insomnia Education Website; n=83, Sleep Healthy Using the Internet.

### Post Hoc Analyses

**Figure 2:**
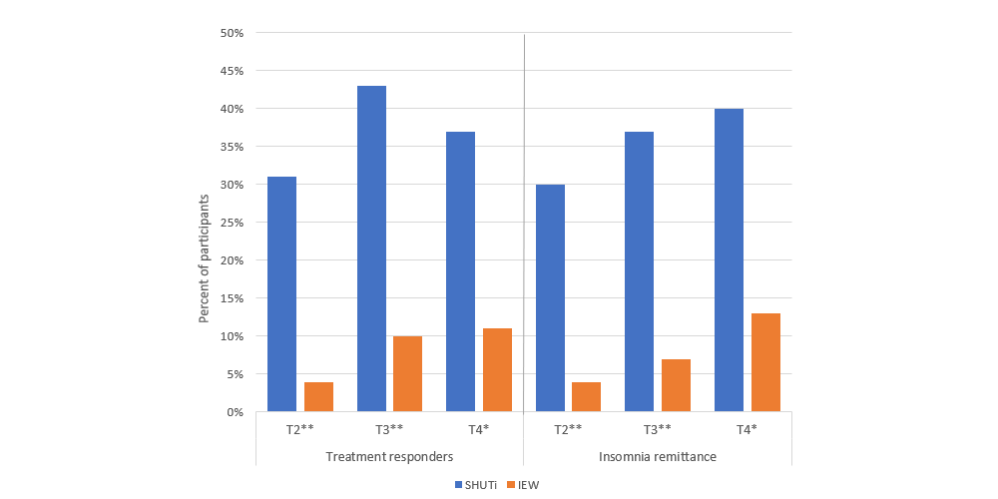
Treatment responders and insomnia remittance by group. Treatment responders are defined as a reduction of >7 points on the Insomnia Severity Index (ISI) between T2, T3, and T4, respectively, and T1. Insomnia remittance is defined as an ISI change from ≥8 at T1 to <8 at T2, T3, and T4. IEW: Insomnia Education Website; SHUTI: Sleep Healthy Using the Internet. *P<.001; **P<.0001.

A significantly greater proportion of the SHUTi group were deemed treatment responders (reduction of greater than 7 points on the ISI) at all time points compared with IEW participants (31% vs 4% at T2; 43% vs 10% at T3, and 37% vs 11% at T4; all *P*<.001; Figure 2). Additionally, a greater proportion of the SHUTi group met criteria for insomnia remittance (change from ≥8 at T1 to <8 on the ISI at T2, T3, and T4) at all time points compared with IEW participants (30% vs 4% at T2; 37% vs 7% at T3, and 40% vs 13% at T4; all *P*<.001; Figure 2).

## Discussion

### Principal Findings

Despite the empirical support for CBT-I as an efficacious first-line treatment for insomnia and evidence that iCBT-I improves insomnia severity with comparable effect sizes to those found in F2F CBT-I, there is a paucity of RCTs examining iCBT-I in veterans. Data from this RCT confirmed the efficacy of SHUTi for the treatment of insomnia in OEF, OIF, and OND veterans, and this is the first study to demonstrate that improvements in insomnia severity are sustained up to 1 year postintervention. SHUTi participants reported significant changes in insomnia severity across all time points compared with IEW participants. These effects were medium-large and consistent with civilian SHUTi trials, an iCBT-I trial [89] in a military sample, and self-guided iCBT-I interventions in veteran samples [32-34]. SHUTi participants also experienced significant improvements in sleep diary parameters compared with IEW participants. Although SHUTi participants experienced a reduction in total sleep time compared with IEW participants, they spent less time in bed and improved their sleep efficiency, which are consistent with CBT-I desired outcomes.

IEW participants completed pre- and postintervention sleep diaries and had access to high-quality, patient-oriented insomnia materials, constituting a more “active” control compared with waitlist designs. For example, behavioral tracking can facilitate awareness of patterns that foster insight and promote behavior change. Given this, the fact that SHUTi participants significantly outperformed IEW participants in reducing their insomnia severity demonstrates the value of metered, interactive, and tailored iCBT-I content. These findings add to the robust empirical data that supports the superiority of CBT-I interventions over approaches such as sleep hygiene education. This study extends those by demonstrating the efficacy of self-administered iCBT-I in a veteran population.

SHUTi participants were also more likely than IEW participants to report significant improvements in health-related quality of life as assessed by the VR-36 during follow-up, though effect sizes were small. These findings suggest that mental and physical health functioning may require sustained improvements in insomnia to ultimately improve functioning. Furthermore, given the many health factors impacting veterans’ health-related quality of life, it is possible that small effect sizes were due to improved sleep but the continued negative impact of comorbid issues. Our findings were consistent with those observed in a military sample [90] but differed slightly from findings reported in a systematic review on F2F CBT-I and functioning [91], suggesting potential differences in the insomnia-functioning association across populations. Although our findings fall below the change typically indicative of clinical significance at an individual level, they are above the cutoff for population clinical significance [79,92]. Paired with the insomnia severity findings, this suggests that health care implementation of iCBT-I could be an especially promising approach to improving veteran outcomes (eg, systems level).

SHUTi participants were more likely to achieve clinically meaningful changes in insomnia severity compared with IEW participants across all time points, consistent with improvements observed at posttreatment by veterans following use of Insomnia Coach [33]. The proportion of treatment responders (31%) and those who achieved remittance (30%) at posttreatment was greater in our SHUTi sample compared with an implementation-focused study of SHUTi in VA primary care (20% responders, 18.6% remitted) [34]. These differences may have been driven by sample characteristics, as participants in this study were younger (mean age of 39.3, SD 7.8 years) than those in the study of Hermes et al [34] (mean age of 67, SD 16.9 years). Furthermore, participants in this study may have been more motivated to engage in the iCBT-I as enrollment originated from self-interest versus provider referral. Despite SHUTi participants achieving clinically meaningful changes in insomnia, responder and remittance percentages were lower than what had been observed in nonveteran SHUTi trials. IEW participants, however, were also less likely to naturally recover or receive benefit from the IEW when compared with nonveteran IEW participants, suggesting that differences may be attributable to methodology and sample clinical complexity. Given that this study was not designed to specifically examine comorbidity, future studies are necessary to investigate how clinical comorbidity impacts iCBT-I engagement and benefit.

For self-administered approaches to be a viable health care option, individuals must be interested in using technology to receive an intervention and effectively engage. OEF, OIF, and OND veterans’ interest in this study was high, with almost 900 veterans reporting interest in approximately 9 months. Engagement was also high, with 58% of SHUTi participants engaging in the intervention to receive an adequate dose (ie, completion of more than 4 cores) [44]. This finding is notable given there was no clinician or research staff support during the intervention, demonstrating that some veterans can achieve positive outcomes from a self-administered iCBT-I without clinician or coaching support.

### Limitations

Although this trial was adequately powered and included a longitudinal design with a high retention rate in an important clinical population, several limitations are important to note. To mitigate primary outcome confounding, there was no ISI inclusion requirement. As such, about one-third of randomized participants reported insomnia severity below the ISI clinically significant threshold. Although CBT-I has been shown to provide benefit in subthreshold insomnia [93], this has not been widely studied in veteran populations and may have resulted in a floor effect. Most participants in this study identified as male, White, and non-Hispanic. Thus, our results may not generalize to veterans with different identities who face additional barriers to accessing medical care. To ensure safety, participants were ineligible if they had untreated sleep apnea (a commonly cooccurring sleep disorder with insomnia) [30,94]; our findings may not generalize to veterans with insomnia and untreated sleep apnea. During the consent process, participants were told that the purpose of the study was to learn whether participation in computerized insomnia programs may reduce insomnia symptoms and improve functioning in veterans (ie, SHUTi and IEW were not described or compared). Although participants were blinded to their treatment allocation, based on their experiences (eg, access to static IEW information vs access to SHUTi, seeking information on ClinicalTrials.gov), they may have speculated that their assigned condition was better or worse than the other. To better understand how participant perceptions of assigned conditions impact outcomes, asking participants what condition they believe they received would be of benefit to future iCBT-I trials. Given the RCT design (ie, no initiated contact during the intervention), we do not have a reliable way to ascertain the duration or engagement in SHUTi or the IEW. Although this design improves the ecological validity of our findings, it limits our ability to determine whether there were moderating factors associated with trajectories of improvement. Future research examining levels of support will be integral in determining CBT-I treatment options (ie, clinician-delivered CBT-I and clinician-supported and self-administered iCBT-I) [33,95-97]. Although participants must have experienced insomnia symptoms for at least 3 months to meet the inclusion criteria, we did not systematically collect information on insomnia symptom duration, sleep medication use, or medical comorbidity; this information would have further characterized the sample and will be important to include in future iCBT-I research. Finally, although commonly used as an important indicator, sleep outcomes were based on self-report, with no objective indicators. Future research examining potential differences in subjective versus objective outcomes following SHUTi will be critical to understanding the mechanisms of change.

### Conclusions

This study is the first known longitudinal RCT comparing an evidence-based iCBT-I to a nonwaitlist control in a veteran population meeting diagnostic criteria for insomnia disorder. In this RCT, we observed significant reductions in the primary outcome of insomnia severity between groups immediately after the intervention and up to 1 year postintervention. Although the secondary outcomes of improved physical and mental health functioning were not detected immediately after the 9-week intervention, SHUTi was efficacious in improving mental health functioning at both follow-up time points and physical health functioning at 6 months. In exploratory analyses, participants randomized to SHUTi experienced significant improvements across sleep parameters. The results of this RCT support the use of a self-administered iCBT-I in OEF, OIF, and OND veterans who meet criteria for insomnia disorder.

## Appendix

Multimedia Appendix 1 Trial protocol.

Multimedia Appendix 2 Exploratory measures.

Multimedia Appendix 3 CONSORT-EHEALTH checklist (V 1.6.1).

## Abbreviations

CBT-I: Cognitive behavioral therapy for insomnia
DSM-5: Diagnostic and Statistical Manual of Mental Disorders, Fifth Edition
EBP: evidence-based psychotherapy
EMA: early morning awakenings
F2F: face-to-face
iCBT: internet-delivered cognitive behavioral therapy
iCBT-I: internet-delivered cognitive behavioral therapy for insomnia
IEW: Insomnia Education Website
ISI: Insomnia Severity Index
MCS: Mental Component Summary
OEF: Operation Enduring Freedom
OIF: Operation Iraqi Freedom
OND: Operation New Dawn
PCL-5: Posttraumatic Stress Disorder Checklist for Diagnostic and Statistical Manual of Mental Disorders, Fifth Edition
PCS: Physical Component Summary
RCT: randomized controlled trial
SHUTi: Sleep Healthy Using the Internet
SOL: sleep onset latency
TIB: time in bed
TST: total sleep time
VA: Department of Veterans Affairs
Veterans SF-36: Short Form Health Survey for Veterans
VHA: Veterans Health Administration
VR-36: Veterans RAND 36-item Health Survey
WASO: wake after sleep onset
