# Supplementary material for: Efficacy of an Internet-Delivered Intervention for Improving Insomnia Severity and Functioning in Veterans: Randomized Controlled Trial
Source: JMIR Ment Health. 2023 Nov 24;10:e50516. doi: 10.2196/50516 (PMC10709797; doi:10.2196/50516)
Supplement: Multimedia Appendix 1 [file mental_v10i1e50516_app1.docx]

| w | *COMIRB Protocol* |  |
| --- | --- | --- |
| COLORADO MULTIPLE INSTITUTIONAL REVIEW BOARDCAMPUS BOX F-490 TELEPHONE: 303-724-1055 Fax: 303-724-0990 | |  |

**Protocol #: 17-0920**

**Project Title: Vets Sleep**

**Principal Investigator: Sarra Nazem, Ph.D.**

**Version Date: 10-18-18**

**I**. **Hypotheses and Specific Aims**:

**Specific Aim**: Determine the efficacy of a prominent computerized cognitive behavioral therapy (cCBT), called Sleep Healthy Using the Internet (SHUTi), for treatment of insomnia among Operation Enduring Freedom (OEF), Operation Iraqi Freedom (OIF), and Operation New Dawn (OND) Veterans. Efficacy will be evaluated using a two group (SHUTi vs. educational website control) longitudinal (four time points) randomized controlled trial (RCT) design.

*Objective 1.1*: Determine if there are significant differences in insomnia symptom reduction and physical and mental health functioning between participants in the two groups.

*Hypothesis 1.1*: Participants randomized to SHUTi will report a significant pre-intervention to post-intervention decrease in insomnia symptoms, and improvement in functioning compared to participants who are randomized to the educational website control.

*Objective 1.2 & 1.3*: Determine whether changes in insomnia symptoms and physical and mental health functioning are maintained six-months and one-year post-intervention.

*Hypothesis 1.2 & 1.3*: Participants randomized to SHUTi will report a significant pre-intervention to six-months and one-year post-intervention decrease in insomnia symptoms, and improvement in functioning compared to participants who are randomized to the educational website control.

*Exploratory Objective 1.4*: Determine whether SHUTi is associated with significant reductions in sleep diary parameters (i.e., sleep efficiency, sleep onset latency, wake after sleep onset) and key variables including suicidal ideation, depressive symptoms, anxiety, and post-traumatic stress disorder (PTSD)).

**II**. **Background and Significance**:

Chronic sleep problems are a major complaint among OEF/OIF/OND Veterans, with rates of insomnia dramatically increasing over the past decade [49-51]. Elevated rates of insomnia are especially concerning given that insomnia may precede functionally impairing symptoms of depressive episodes, post-traumatic responses, and self-directed violence (SDV) [2,52,53]. Furthermore, even after resolution of comorbid symptoms, insomnia symptoms often remain at clinical levels, and continue to impact functioning [54]. Thus, it is imperative that individuals suffering from insomnia receive evidence-based therapies (EBT) that reduce insomnia and associated comorbid symptoms, improve quality of life, and increase function.

Despite the high prevalence of insomnia, only a small percentage of individuals with insomnia actually receive any type of treatment for their symptoms [55]. Cognitive Behavioral Therapy for Insomnia (CBT-I) has quickly emerged as one of the most efficacious treatments for insomnia. In 2005, the National Institutes of Health (NIH) State-of-the-Science conference on insomnia identified CBT-I as a first-line treatment given that CBT-I was found to be as effective as prescription medications for the short-term treatment of insomnia. Consequently, CBT-I has emerged as the “treatment of choice” for managing sleep/wake complaints [15], with evidence suggesting that 70-80% of individuals obtain benefit from the intervention [56]. The efficacy of CBT-I has been established in numerous studies and outlined in three meta-analyses [56-59].

Despite the widespread data in support of CBT-I, many VA facilitates do not have sufficient access to trained CBT-I providers. Due to lack of access to trained clinicians, coupled with the increasing demand for services from returning OEF/OIF/OND Veterans, the need for evidenced-based sleep interventions will continue to intensify. Thus, despite the VA’s efforts to disseminate CBT-I, there remains a necessity to expand access to CBT-I above and beyond what can be delivered by CBT-I trained providers. Evidence for the positive effects of CBT-I, along with access issues, suggests the need to incorporate innovative methods, such as cCBTs, for delivery of CBT-I. Existing web-based cCBTs offer a potentially efficacious means of delivering insomnia treatments by providing the opportunity to engage in an EBTs via computers. From both a policy and clinical perspective, cCBTs have garnered attention due to their potential to overcome barriers to treatment, including cost, scheduling and travel demands, stigma, and lack of trained EBT clinicians [25]. Furthermore, leveraging technology to meet treatment demands is consistent with service delivery models based upon stepped care principles [60].

The SHUTi program is based upon the CBT-I model, consisting of the same behavioral, educational, and cognitive treatment components underlying CBT-I and has been found to be efficacious for the treatment of insomnia [40-44]. In the first SHUTi pilot trial [40], 44 civilian adults with primary insomnia were randomly assigned to either SHUTi or a wait-list control group. Prospectively collected sleep diary data showed significant improvements for SHUTi users in sleep efficiency, nighttime awakenings, and total sleep time; whereas the control group showed no significant changes. SHUTi participants also showed clinical improvement in insomnia severity, with 73% of SHUTi users falling into the “no insomnia” category after treatment; no control participants met this criterion. Positive gains were also maintained for SHUTi participants at six months. In addition, although SHUTi was not specifically designed to target comorbid symptoms, significant improvements were found in mood (e.g., depressive and anxious affect), overall health (e.g., fatigue), and quality of life [41]. A recent RCT by Christensen and colleagues, demonstrated that SHUTi, compared to an Internet-based placebo control group, was significantly associated with lower depression symptoms at six-weeks and six-months compared to the control group in a sample of Australian Internet users with insomnia and depression symptoms that did not meet criteria for major depressive disorder [61]. In sum, data from several rigorous clinical trials, including a variety of civilian patient groups, consistently show significant and clinically meaningful improvements in sleep outcomes and co-occurring symptoms with SHUTi.

Despite the growing evidence base for SHUTi, no RCT of SHUTi has been conducted with Veterans or military personnel. Research demonstrating the efficacy of SHUTi among military and Veteran populations is essential to meeting treatment demands and improving the health of Veterans. Given empirical evidence supporting the association between insomnia and comorbid conditions, SHUTi may offer an invaluable resource to address the needs of Veterans and the Veterans Health Administration (VHA).

**III**. **Preliminary Studies/Progress Report:**

As noted above, several researchers have provided efficacy data supporting SHUTi as a treatment intervention that is associated with reductions in insomnia and comorbid symptoms, yet no RCT of SHUTi has been conducted within a Veteran population. No prior research on SHUTi has been conducted by this team at the ECHCS.

**IV. Research** **Methods**

**A. Outcome Measure(s):**

***Screening Measure:***

*Screening Form:* A screening form will be used to assist in the assessment of eligibility criteria (e.g., OEF/OIF/OND Veteran, eligible to receive care at DVAMC, insomnia diagnosis).

***Primary Outcome Measure:***

*Insomnia Severity Index (ISI)* [46]*.* The ISI is a seven-item index of subjective sleep difficulty. Respondents provide information on difficulty with sleep onset, sleep maintenance, and early morning awakenings, as well as interferences with daytime functioning, how noticeable sleep problems are to others, distress caused by problematic sleep, and overall sleep satisfaction. Total scores range from 0 to 28 with higher scores indicating greater insomnia severity. The ISI is a reliable, valid measure that can be delivered through the Internet [62] and is sensitive to changes in insomnia treatment studies [46]. The suggested guidelines for interpretation are: 0-7 = no clinically significant insomnia; 8-14 = subthreshold insomnia; 15-21 = clinical insomnia of moderate severity; 22-28 = severe clinical insomnia.

***Secondary Outcome Measure:***

*The Veteran’s SF-36 (VA SF-36)* [63]: The VA SF-36 is the primary measure of health-related quality of life used within VA populations, built on the Medical Outcomes Study (MOS) SF-36 [64]. The Veteran’s SF-36 measures eight concepts of health, with two summary scores: physical component summary and mental component summary.

***Additional Measures:***

*Adult Suicidal Ideation Questionnaire (ASIQ)* [47]. The ASIQ is a 25-item self-report instrument designed to estimate a respondent’s current level of suicidal ideation. Each ASIQ item is rated on a 7-point scale indicating the frequency of each item over the past month. The ASIQ has high internal consistency and test-retest reliability in clinical and nonclinical samples [65].

*Beck Anxiety Inventory (BAI)* [48]. The BAI is a 21-item, self-report rating inventory that measures subjective, somatic, and panic-related symptoms of anxiety.

*Beck Depression Inventory II (BDI-II)* [66]. The BDI-II is a 21-item, self-report rating inventory that measures characteristic attitudes and symptoms of depression.

*Follow-Up Questions:* During the follow-up assessments, all participants will be asked about whether they have made any changes to sleep medications and sought sleep treatment in addition to brief inquiries about demographic changes (i.e., marital status, employment status, student status, current homelessness). Participants that received SHUTi will be asked four brief open-ended questions designed to collect qualitative information on how participation in SHUTi has affected the participant, the most helpful aspect of participating, and ways to improve SHUTi.

*Internet Evaluation and Utility Questionnaire* [43]: The Internet Evaluation and Utility Questionnaire measures participants’ experiences and perceptions of an Internet intervention. The constructs measured include items on ease of use, convenience, engagement, enjoyment, layout, privacy, satisfaction, and acceptability. Additional items assess perceptions of web program material in terms of usefulness, comprehension, credibility, likelihood of returning, mode of delivery, and helpfulness.

*Internet Impact and Effectiveness Questionnaire* [43]: The Internet Impact and Effectiveness Questionnaire measures participants’ perceptions of an Internet intervention in terms of the program’s effectiveness in resolving their targeted health condition. Perceived impact is measured in terms of helpfulness, knowledge gains, treatment effectiveness for self and others, long-term effectiveness, quality of life, mood, physical activity, family and peer relationships, social activity, school/work attendance and performance, treatment implementation, goal-orientation, confidence in ability to manage condition, relapse prevention, and service reduction.

*Life Experiences Survey (LES):* Based upon the Holmes-Rahe Life Change Index Scale [67], the LES queries participants about a variety of life events (e.g., death of a close friend, loss of child). Participants will be asked to indicate yes or no as to whether any of these life events have occurred since the last study assessment as a way to characterize major life events that may be associated with long-term changes in sleep patterns.

*Military Suicide Research Consortium (MSRC) Common Data Elements (CDE).* The MSRC CDE is a composite measure of several items taken from commonly utilized self-report assessments. The CDE are required to be administered to all participants by the funding agency.

*Military Suicide Research Consortium (MSRC) Demographics:* Required by the funding agency, this demographics form will collect information (e.g., gender, race, education, military history) to describe key demographic and military participant characteristics.

*Morningness/Eveningness Questionnaire (MEQ)* [68]: The MEQ is a 19-item self-report instrument designed to assess an individual’s chronotype: evening, intermediate, or morning. Information about participant chronotype will help determine whether certain chronotypes may be more or less sensitive to conventional insomnia treatment.

*Posttraumatic Stress Disorder Checklist for DSM-5 (PCL-5)* [69]. The PCL-5 is a psychometrically sound self-report measure that assesses the severity of PTSD symptoms in the past month.

*Sleep Diary* [70]. A Sleep Diary includes assessment of 10 standard sleep parameters: time to fall asleep; number and length of awakenings at night; time of awakening and arising from bed in the morning; length of naps; subjective sense of how refreshed the participant felt upon awakening as well as how sound they sleep during the night; and amount of medication and alcohol used as a sleep aid. Data suggest that sleep diary assessments are a valid and reliable way to collect information on sleep parameters. They have also been found to provide a more comprehensive understanding of sleep patterns than can be achieved using objective measures of sleep, such as polysomnography, which is typically administered for one to two nights only [71]. Sleep diaries will provide information on over the counter and prescribed sleep medication use.

*Revised Narrative Evaluation of Intervention Interview (NEII)* [72]: The NEII, slightly revised by this study team to be applicable to SHUTi, is an assessment tool designed to help participants evaluate and describe the process and outcome of an intervention. This qualitative feedback will be used to inform future implementation efforts.

*Revised Reasons for Termination Questionnaire* [73]: The Reasons for Termination Questionnaire, slightly revised by this study team to be applicable to SHUTi, asks participants to indicate reasons why (e.g., time problems, problems improved and no longer felt a need for intervention) they stopped participating in the intervention. Participants are then asked to rate the amount of influence each reason had on the decision to stop participating.

**Table S1.** Measures, time to completion, and purpose of study measures.

| **Measure** | **Time** | **Purpose** |
| --- | --- | --- |
| **Phone Screening** | | |
| Screening Form | 5-20 | Determine eligibility for the study |
| **Baseline Assessment (Time 1): 100 minutes** | | |
| Insomnia Severity Index (ISI) | 5 | Assess insomnia symptoms |
| Veteran’s SF-36 | 15 | Assess health-related quality of life |
| MSRC Demographics | 5 | Collect participant demographics |
| Adult Suicidal Ideation Questionnaire (ASIQ) | 10 | Assess past-month suicidal ideation |
| PCL-5 | 5 | Assess PTSD symptoms |
| Beck Depression Inventory-II (BDI-II) | 5 | Assess depressive symptoms |
| Beck Anxiety Inventory (BAI) | 5 | Assess anxiety symptoms |
| Morningness/Eveningness Questionnaire | 10 | Assess factors impacting circadian rhythm |
| MSRC Common Data Elements | 40 | Study sponsor measure – abbreviated self-report items |
| **Post-Intervention Assessment (Time 2): 85-120 minutes** | | |
| Insomnia Severity Index (ISI) | 5 | Assess insomnia symptoms |
| Veteran’s SF-36 | 15 | Assess health-related quality of life |
| Adult Suicidal Ideation Questionnaire (ASIQ) | 10 | Assess past-month suicidal ideation |
| PCL-5 | 5 | Assess PTSD symptoms |
| Beck Depression Inventory-II (BDI-II) | 5 | Assess depressive symptoms |
| Beck Anxiety Inventory (BAI) | 5 | Assess anxiety symptoms |
| Revised Reasons for Termination Questionnaire | 5* | Assess reasons for non-completion of SHUTi |
| Revised NEII | 15** | Qualitative feedback designed to inform future implementation efforts |
| Internet Evaluation and Utility Questionnaire | 5** | Feedback on experiences and perceptions of SHUTi |
| Internet Impact and Effectiveness Questionnaire | 10** | Feedback on perceptions of SHUTi’s effectiveness |
| MSRC Common Data Elements | 40 | Study sponsor measure – abbreviated self-report items |
| **6-Month Follow-Up Post-Intervention Assessment (Time 3): 95-100 minutes** | | |
| Insomnia Severity Index (ISI) | 5 | Assess insomnia symptoms |
| Veteran’s SF-36 | 15 | Assess health-related quality of life |
| Adult Suicidal Ideation Questionnaire (ASIQ) | 10 | Assess past-month suicidal ideation |
| PCL-5 | 5 | Assess PTSD symptoms |
| Beck Depression Inventory-II (BDI-II) | 5 | Assess depressive symptoms |
| Beck Anxiety Inventory (BAI) | 5 | Assess anxiety symptoms |
| Follow-Up Questions – SHUTi | 5** | Feedback on experiences and perceptions of SHUTi |
| Follow-Up Questions – Sleep and Demographics | 5 | Information on changes in sleep medication/treatment and demographics |
| Life Experiences Survey | 5 | Information on life changes since last assessment |
| MSRC Common Data Elements | 40 | Study sponsor measure – abbreviated self-report items |
| **1-Year Follow-Up Post-Intervention Assessment (Time 4): 95-100 minutes** | | |
| Insomnia Severity Index (ISI) | 5 | Assess insomnia symptoms |
| Veteran’s SF-36 | 15 | Assess health-related quality of life |
| Adult Suicidal Ideation Questionnaire (ASIQ) | 10 | Assess past-month suicidal ideation |
| PCL-5 | 5 | Assess PTSD symptoms |
| Beck Depression Inventory-II (BDI-II) | 5 | Assess depressive symptoms |
| Beck Anxiety Inventory (BAI) | 5 | Assess anxiety symptoms |
| Follow-Up Questions – SHUTi | 5** | Feedback on experiences and perceptions of SHUTi |
| Follow-Up Questions – Sleep and Demographics | 5 | Information on changes in sleep medication/treatment and demographics |
| Life Experiences Survey | 5 | Information on life changes since last assessment |
| MSRC Common Data Elements | 40 | Study sponsor measure – abbreviated self-report items |

*Assessment administered only to participants randomized to SHUTi that did not complete all 6 cores. **Assessments administered only to participants randomized to SHUTi.

Note: Sleep diaries will be completed two weeks before baseline assessment (Time 1) and during the two weeks after post-intervention assessment (Time 2). Participants will also complete the ISI and sleep diaries throughout the intervention. If a SHUTi participant contacts the study team indicating that they would like to withdraw from the study, the participant will have the opportunity, if willing, to provide responses to the Revised Reasons for Termination Questionnaire, via phone.

1. **Description of Population to be Enrolled:**

Participants will be recruited from populations of Veterans receiving or eligible to receive physical and mental health care within the VA Eastern Colorado Health Care System (ECHCS). We will enroll up to 250 eligible Veterans who meet the study inclusion/exclusion criteria described below.

***Recruitment***

Recruitment strategies will encompass both facility- and Veteran-based recruitment methods to maximize potential recruitment of OEF/OIF/OND Veterans. For facility-based recruitment, the research team may work with providers within the ECHCS to enlist their assistance with recruitment. Research team members may attend team meetings and/or brief staff about the study. Staff may pass along the approved flyer to Veterans who may be interested. Veterans may also see our approved flyer within the facility. In addition to recruiting from facilities within the ECHCS, the research team will recruit Veterans in the community who are eligible to receive VHA care. Approximately 60% of Veterans do not seek care within VHA [74]. Recruiting members of this population is expected to facilitate overall participant accrual, increase participation by women and minorities, and maximize the generalizability of findings. The Rocky Mountain MIRECC collaborates with partner organizations within the community to enhance representation of Veterans not seeking VHA care. In addition, the study will be advertised on the internet via MIRECC affiliated websites, and approved letters describing the study and inviting participation will be sent to those who have participated in past studies and have been added to the Rocky Mountain MIRECC research data repository.

Given that some Veterans may have limited contact with Veteran Affairs Medical Centers (VAMCs), and as a result be unlikely to learn about the opportunity to participate in the study through normal means (e.g., advertisements within the VA Medical Center, provider referral), we will also engage in Veteran-based recruitment strategies. Veterans will be identified in the Corporate Data Warehouse (CDW) for possible eligibility for this study. CDW is a national repository comprising data from several VHA clinical and administrative systems, organized into a collection of data domains. Individuals with ICD-9 and ICD-10 codes of interest (e.g., G47.00) will be identified from various tables within the CDW that correspond to diagnoses given at inpatient/outpatient VA visits and fee-basis visits. Similarly, other insomnia related variables within the electronic medical record or CDW (e.g., notes associated with sleep consults or treatment, medication and problem lists, service connection, mental health assistant assessments) may also be used to identify individuals for recruitment purposes. Demographic data within the CDW and the Veterans Business Administration (VBA) will also be used to determine eligibility. Names and addresses of Veterans who may be eligible will be pulled from the CDW and/or VBA for recruitment purposes. An initial invitation may be sent by U.S. mail from the study PI, to participate in the study with a pre-stamped and addressed ‘Refusal Response Card’. If the potential participant contacts the study team and expresses interest in participating, he/she may then be screened for possible enrollment into the study. The refusal response card will not include any PHI, nor the study name or Veteran’s name. So that the research staff is able to identify a Veteran who would no longer like to be contacted, the refusal response card will include a randomly-generated unique identifier number so that the research team can link the person’s response with their identity using the unique identifier. The refusal response card only states, “I do not wish to participate in this study.” If the potential participant returns the refusal response card, that individual’s name will be flagged as ‘do not contact.’ One additional letter inviting participation may be sent if the participant has not expressed either interest in participating, or no interest in participating by returning the refusal response card, within 4 weeks of the initial letter being sent out. Potential participants recruited after obtaining their name and address and relevant diagnoses from the CDW and/or VBA will be formally consented prior to any study activities taking place.

***Inclusion/Exclusion Criteria***

**Inclusion Criteria:**

1. Age between 18 and 55
2. History of deployment in the Global War on Terror
3. Eligible to receive care through VA ECHCS
4. Reliable access to the Internet
5. English speaking
6. Able to provide informed consent
7. Current insomnia diagnosis as defined by Diagnostic and Statistical Manual of Mental Disorders 5 (DSM 5) criteria [45]

**Exclusion Criteria:**

1. Currently enrolled in/participating in other intervention research studies
2. Other untreated sleep disorders (e.g., sleep apnea, periodic limb movement)
3. Currently receiving formal psychological treatment for insomnia (not including sleep medications)
4. Past 3 month change in schedule and/or dosage of medications that are designed to improve/impact sleep
5. History of Bipolar Disorder (with manic episodes), Schizophrenia, Schizoaffective Disorder, or a Psychotic Disorder
6. Untreated seizures or seizure disorder
7. Physical illness that is active, unstable, degenerative, and/or progressive
8. Currently pregnant or plan to become pregnant in the next 6 months
9. Irregular work schedule, shift work, and/or life changes (e.g., new baby) interfering with regular sleep patterns
10. Significant cognitive impairment, as determined by chart review and/or during screening, that would interfere with ability to engage in SHUTi
11. Current non-alcohol Substance Use Disorder, excluding Cannabis Use Disorder, as determined by chart review and/or self-report screen of drug use (> 1 time) in past 3 months
12. Current alcohol use diagnosis, as determined by SCID 5 module, in the past 3 months
13. **Study Design and Research Methods**

**Figure S1.** Consolidated Standards of Reporting Trials (CONSORT) diagram.


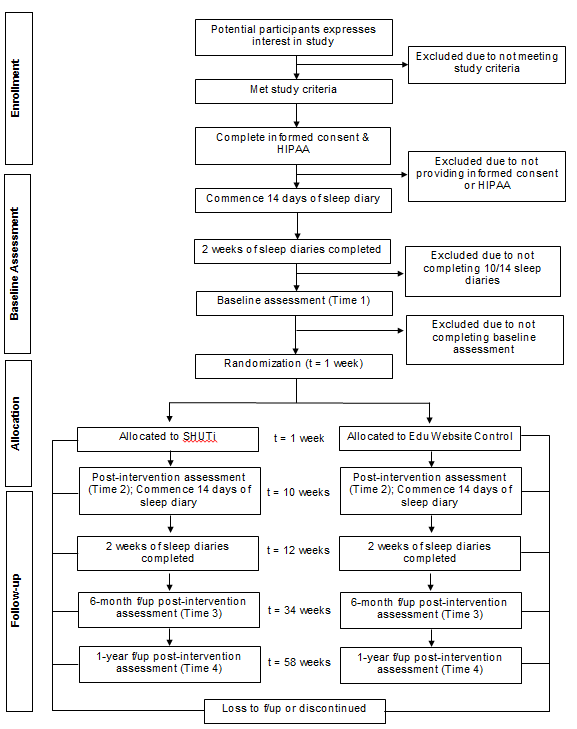


***Screening Examinations & Procedures***

A Veteran expressing interest in participating in this study will participate in a screening process to assess for study inclusionary/exclusionary criteria. A waiver of consent (including waiver of written documentation) and a HIPAA Waiver of Authorization will be requested (see screening script with verbal consent) for the screening portion of the study. This screening process, which will be conducted by a member of the research team, may include a review of medical records and/or a brief phone interview. During the screening, a screening form will be used to facilitate acquisition of basic information to assist in determination of the inclusion/exclusion criteria. The screening form will not contain PHI. However, personal information is required for participant contact and electronic medical record review for confirmation of medical/psychiatric history. Screening data will be kept separate from other study data. Once interest in the study has been conveyed and screening procedures have been completed, a member of the research team will explain the intervention/assessment procedures to the participant. If the participant is eligible and agrees to participation in the study, the research team member will continue on with the consent process. If necessary, another phone appointment may be scheduled to complete the consent process.

***Consent Procedures***

All study procedures will be conducted via the telephone and the Internet. For this reason, we are requesting a wavier of documentation of consent and HIPAA. A consent process will still occur over the telephone with all eligible participants.

**Assessing ability to provide consent**. Individuals interested in participating will be assessed for their ability to provide informed consent, based on their responses to the following questions:

1. What are you being asked to do if you participate in this study?
2. Finish this sentence – The purpose of this study is to find out…
3. True or False: After beginning this study, you can decide to not continue at any time, without penalty.
4. What should you do if you have questions about this study?
5. Who should you call if you feel you have been harmed in this study?
6. What are the risks of being in the study?
7. What are the benefits of being in the study?

If the individual is able to adequately answer these questions and decides to participate, verbal assent will be obtained. Individuals who are not able to adequately respond to the questions above will be excluded from participation. Participants will be provided with ample time to ask questions.

Following the consent process, including the consent questions, potential participants will be asked to verbally provide their assent. Participants will be able to view the postcard consent upon their initial login to the study website.

Enrollment into the study will be documented in the participant’s electronic VA medical chart.

***Assessment & Intervention Procedures***

Following the consent process, while on the telephone, the research team member will provide the participant with the web address for the B*e*Health Solutions Vets Sleep registration website. The participant will visit this website to register. Once registered, study staff will confirm the participant’s registration, which will then send an automated email to the participant inviting them to the B*e*Health Solutions Vets Sleep study-specific website. At this website, participants will have the ability to review the postcard consent and set up their user profile which will then allow the participant to establish their personal login and password.

The procedures above will ensure that participants are correctly accessing the web-based platform that has been specifically designed for the Vets Sleep research study (i.e., not accessing any B*e*Health Solutions/Pear Therapeutics SHUTi products that are meant for the public domain/commercial entity). As part of the grant funding supporting this research, Denver Research Institute (DRI), the non-profit organization managing the grant, has a contract with B*e*Health Solutions. As part of this contract, B*e*Health Solutions/Pear Therapeutics will provide 113 SHUTi licenses and 113 educational website control licenses.

*Sleep Diary Pre-Assessment:* After enrollment in the study, participants will then be provided instructions on how to complete a two-week sleep diary assessment online via the study website. Participants will be informed that they must complete 10/14 sleep diaries in order to move forward in the study. For each sleep diary (1/day), participants will be compensated $1, for a total of $14.

*Baseline Assessment:* After completing at minimum 10/14 days of sleep diaries, participants will then be invited to complete their baseline assessment (see Table S1 above). Participants will be compensated $50 for baseline assessment. Participants may receive a phone call or email reminding them to complete the baseline assessment.

*Random Allocation and Concealment:* Random allocation will be conducted by the Study Coordinator after baseline assessment completion; randomization will be stratified by suicide attempt history. The randomization scheme will be produced using Statistical Analysis System (SAS) PROC PLAN with a specified seed and random block sizes, prior to any recruitment and will be conducted by Dr. Forster. To ensure concealed allocation, participants will be designated by an identification (ID) number. ID numbers will only be forwarded to the Study Coordinator, who will randomly allocate participants based on the randomization scheme. In this way, only the Study Coordinator (not the participant, blind assessor, or other research personnel involved in data acquisition) will know condition assignment.

*Intervention:* Following the baseline assessment, up to 226 Veterans will be randomized to SHUTi (n = 113) or the educational website control (n = 113). These interventions will run for nine weeks.

*SHUTi*: SHUTi is a self-guided (i.e., automated), interactive, and tailored web-based program modeled on the primary tenants of face-to-face CBT-I. Intervention content is delivered through six “cores.” Users will obtain access to a new core based on a time and event-based schedule (e.g., seven days after completion of previous core); ISI is administrated in tandem with completion of each core. This schedule is consistent with the recommendation from a task force commissioned by the Academy of Sleep Medicine, which deemed that an average of six to eight sessions constitutes “adequate treatment exposure” [75]. The SHUTi program relies on user-entered online sleep diaries to track progress and to tailor treatment recommendations (i.e., assign a “sleep restriction” window). Each core acts as an online analog for the weekly sessions typically used when delivering CBT-I in a face-to-face format, following the same general structure: 1) core objectives (what will be learned and why this information is important); 2) review of previous week’s homework and sleep diary data; 3) new intervention material; 4) assignment of homework (treatment strategies for the coming week); and, 5) a summary of the core’s main points. Intervention content is enhanced through a variety of interactive features, including personalized goal-setting, graphical feedback based on inputted symptoms, animations/illustrations to enhance comprehension, quizzes to test user knowledge, patient vignettes, and video-based expert explanation. Automated emails are sent to encourage program adherence which is an important component to facilitate adherence and outcomes in self-guided Internet delivered interventions, especially for patients with comorbid or elevated symptoms [29].

*Educational Website Control:* The educational website control used in this RCT has been previously developed by University of Virginia researchers. When developing the educational website control, researchers conducted a thorough online review to determine what constituted “treatment as usual” for patient education websites targeting insomnia. Informed by this review, the educational website control was designed and includes the following: 1) content presented all at once; 2) treatment information about insomnia symptoms, diagnosis and differential diagnosis of insomnia, etiology, the natural history of insomnia and its prognosis, and cognitive-behavioral treatment strategies; 3) printable documents (including sleep diaries and insomnia severity assessments); 4) a FAQ section which provides answers to frequently encountered patient problems; and, 5) automated email prompts to encourage users to return to the site. Non-automated individual patient inquiry elements, such as forums and “ask the expert,” are not included as these can confound the obtained content (as different users may or may not receive helpful content, if anything at all). As indicated above, a detailed FAQ section is included, which parallels this type of content but in a more systematic way.

*Post-Intervention Assessment:* Following the intervention period, all allocated participants will be contacted to complete the post-intervention assessment. Participants will receive a phone call or email reminding them to complete the post-intervention assessment online via the web-based platform. The study team member coordinating the assessment will be blind to the participant’s group membership. To ensure that both the blind assessor and study participant do not gain information relevant to their group assignment, the blind assessor will remind the participant at the beginning of the reminder call to not discuss information about their web-based participation as this information may signal whether the participant received SHUTi or the educational website control. After each post-intervention assessment reminder, the blind assessor will be asked to record whether the participant broke the blind. Please see Table S1 for a list of all post-intervention assessments. Participants will be compensated $50 for the post-intervention assessment.

*Sleep Diary Post-Intervention Assessment*. After completion of the post-intervention assessment, participants will be asked to again complete two weeks of sleep diaries using a web-based interface. For each sleep diary (1/day), participants will be compensated $1, for a total of $14.

*Six-month and One-year Post-Intervention Follow-up Assessments.* All allocated participants will be contacted to complete follow-up assessments using the procedure outlined in the post-intervention assessment. Please see Table S1 for a list of all follow-up assessments. Participants will be compensated $40 at each follow up. Participants may receive a phone call or email reminding them to complete the follow-up assessment. Participants will receive a reminder card about their upcoming follow-up assessment at roughly three and nine months since enrollment.

**D. Description, Risks and Justification of Procedures and Data Collection Tools:**

All participants will be provided with ample time to ask questions prior to enrolling in the study. As noted above, a series of questions will assess participants’ understanding of the study in order to ensure that participants have adequately comprehended the critical information (e.g., risks/benefits, voluntary nature) and are able to provide informed consent. Participants will be clearly informed that the study is voluntary and that they can withdraw from the study at any time, with no penalty. Additionally, all research staff have been trained in human subjects research.

**Adverse Events**

Significant adverse events are not expected. For the purpose of this study, adverse events are defined as symptoms reported by participants that are directly related to participating in the study. The PI will be responsible for monitoring adverse events and will regularly review data for safety monitoring to ensure adherence to the protocol and standard operating procedures regarding participant safety. All adverse events will be reported to COMIRB within five days of learning of such events. Due to the limited risks of participating in this study, an external Data Safety Monitoring Board will not be appointed. Furthermore, any participants who reports adverse events may be contacted by Dr. Nazem to discuss resources and care that the VA can provide.

**Risks**

The anticipated risks and discomfort associated with research procedures are not greater than those that would be ordinarily encountered during routine clinical care or psychological assessment. Additionally, many Veterans who participate in research of this nature report positive experiences. However, there is potential risk that the subject matter of the study assessments may cause participants to feel uncomfortable or upset. Consequently, the voluntary nature of the survey will be emphasized during recruitment and informed consent. Additionally, although we are not targeting a high-risk population for recruitment in this study (i.e., there is no inclusion criteria regarding suicidal ideation/attempt), resources (e.g., Veterans Crisis Line; information on how to access emergency care; the PI’s phone number) will be provided to all Veterans in the consent form and online platform, in case participants wish to speak to someone about their participation in the research study or about VA resources; these safeguards will help to mitigate this potential risk.

Participants randomized to SHUTi may experience increased tiredness due to restricted time in bed (this is part of the behavioral treatment for insomnia). To minimize the risk associated with sleep restriction, the SHUTi platform does not recommend participants restrict sleep fewer than five hours. The initial assigned sleep window is also comparable to how much total sleep the participant gets (based on individualized sleep diary data), but the sleep time is restricted to a smaller window. For example, rather than allowing a participant to get 6 hours of sleep while spending 8 hours in bed, a participant will be asked to spend only 6 hours in bed. To further minimize risks associated with sleep restriction, we will instruct participants that they may contact us if they have significant concerns. Based on the situation, we will provide recommendations, including changing their sleep window or incorporating naps. Participants will also be told to avoid operating a car or other heavy machinery when they feel tired. As needed, we will instruct participants to contact their primary care provider or seek professional help at a sleep clinic.

There is also a potential risk of loss of confidentiality and/or privacy. However, a number of protections will be put in place to maintain the confidentiality of records and reduce risks to privacy (see “Data Security and Storage” section below) and this risk is not greater than what is encountered in everyday life activities that require the physical or digital transmission of information. Furthermore, the web-based platforms used in this study all have industry standard safeguards. Moreover, while there is some risk associated with participating in research and disclosing sensitive information, consenting research participants typically agree that the benefits outweigh risks of this nature, and only those who agree to participate after being informed of the potential risks will be included.

**Benefits**

The primary benefit of this study is generalizable knowledge to help better understand whether SHUTi may be an efficacious cCBT for delivery of CBT-I in a Veteran population. If efficacious, this delivery method may help improve Veteran access to a less expensive and EBT for insomnia. It could potentially reduce health disparities by providing access to an EBT for Veterans that seek care at facilities where CBT-I is not available and for Veterans living in rural areas where access to care and treatment involves significant barriers.

Participants randomized to the educational website control and SHUTi may receive the following benefits: knowledge about the diagnosis and symptoms of insomnia, its etiology, methods of monitoring sleep, and general treatment strategies. Participants randomized to SHUTi will receive an interactive intervention that may lead to the reduction of insomnia and comorbid symptoms.

**Safety Monitoring**

Based on the topic of this study, it is not believed that participating in the study places participants at increased risk. However, some participants may be at risk for suicidal ideation due to factors beyond the study and participants will be asked about several suicide risk factors during assessments. There are no clinical cutoffs for the Adult Suicidal Ideation Questionnaire that indicate definite or imminent risk for suicide and no total score is associated with an indication for immediate treatment. During the consent process, all participants will be informed that study team members will not be actively monitoring assessment responses and that immediate clinical needs should be addressed by calling the Veteran’s Crisis Line or 911. The Veterans Crisis Line contact information will be embedded in the web-based platforms as a resource for Veterans who may need additional support, regardless of their score on any specific assessment. From a scientific perspective, it is also important to not provide active outreach/resources to some participants (based on potential total scores) as this could influence the nature of the intervention being provided and could interfere with our ability to determine whether or not the interventions delivered (SHUTi or Educational Website Control) were associated with decreases in suicide risk (Exploratory Objective 1.4). Notably, there is no evidence to suggest that asking about suicidal ideation increases risk for suicide.

***Data Security & Storage***

B*e*Health Solutions/Pear Therapeutics, the companies that will be hosting the study platforms, employs a secure browsing connection that includes SSL certificates that are Transport Layer Security (TLS) 1.2 utilizing strong key exchange (ECDHE_RSA with P-256) and strong cipher (AES_128_GCM) components. In terms of encrypted storage, B*e*Health Solutions/Pear Therapeutics AWS EBS drives are encrypted with industry standard AES-256 algorithms. Furthermore, B*e*Health Solutions/Pear Therapeutics employs a high-level architecture to separate identifying and non-identifying data: one private server is configured behind a firewall where secured data resides; only individuals with approved access onsite are able to connect access this server; a second server is utilized to maintain the front-end Web system so that participants offsite can access the system. Data submitted by participants are captured and then transferred to the private secure server. Participants only need a user ID, password, and email to register and receive study reminder emails. Despite the many industry-approved standards noted above and the high-level architecture of B*e*Health Solutions/Pear Therapeutics’ platform, study-related information (i.e., responses to assessments) cannot be completely guaranteed. This risk will be thoroughly outlined during the consent process.

Minimizing risks pertaining to data security will be achieved by employing safeguards built into the framework of the VA Office of Research and Development (R&D). The PI and data manager will consult with the ECHCS VA Privacy Officer and Information Security Officer through VA (R&D) on data security matters. All data captured within the web-based portals will be transferred to the VA. All data received will be stored within the VA firewall. The data can only be retrieved from within the VA network and will be user restricted so that only members of the research team can access the data. This data will not be kept on laptops that do not meet the VA standards for encryption, anti-virus protection, and firewall security. Data will not leave the VA network except as a deidentified aggregate datasets.

A data use agreement (DUA) with the study sponsor will be utilized to oversee all data sharing that is required by the study sponsor.

Procedures designed to maintain confidentiality will include formal training sessions for all study personnel in the importance of confidentiality and procedures to be followed, as well as formal mechanisms for limiting access to all information that can link data to individual participants. Dr. Nazem, the VA R&D office, and the VA Privacy Officer will be responsible for working with the project staff to ensure the integrity of adherence to patient confidentiality.

**E. Potential Scientific Problems:**

There is a potential that recruitment of participants will be slower than necessary to reach our

goal of 226 participants randomized. If this is the case, we will submit a protocol amendment to change recruitment strategies and our methodological approach. Attrition may be another potential scientific problem. We have estimated 25% attrition in our study design, which is a conservative estimate given that prior RCTs of SHUTi have reported pre-to post-assessment attrition rates from 0-12%, to help reduce the influence of attrition. To improve retention throughout the trial and maximize data collection at designated assessment time points, we will also request that participants provide us with contact information of family and/or friends who would be privy to changes in participant contact information after informed consent; this strategy has improved retention in previous MIRECC studies. Reminder cards will be utilized during follow-up periods to remind participants about study enrollment and to encourage them to contact us in the periods between assessments if contact information has changed.

***Data Integrity***

Dr. Nazem will work with others members of the research team to ensure the integrity of the data by overseeing the monitoring of data collection. Monitoring data collection will entail quarterly checks of all participant data. Designated team members will work with the VISN 19 MIRECC Data Core to check data for completeness and accuracy to minimize data collection errors (or to identify “problem areas”).

**F. Data Analysis Plan:**

***Sample Size***

Power was based off of the nine outcomes comprising Specific Aim 1, Hypothesis 1.1. Given these nine outcomes (insomnia symptom severity based on the ISI, physical and mental component summary scores on the Veteran’s SF-36), a Bonferroni correction was used and we assume an alpha level of 0.0167. We additionally assume a two-sided test of hypothesis and used the two-sample t-test procedure in PASS v13.0.11 to calculate power. Using any pair power, and the Horn sequential procedure121-122 to control the familywise error rate, with a final sample of at least 170 completing the post-intervention assessment (85 per group), we have 80% power to detect an effect size of 0.6 (medium effect size) for each outcome. As we assume an attrition rate of 25%, 226 participants will be randomized to achieve at least 85 per group completing the post-intervention assessment.

***Analysis Plan***

All analyses will assume a two-sided test of hypothesis and a significance level of 0.05, unless otherwise noted. Preliminary descriptive and graphical analyses (e.g., boxplots, scatterplots) will be used for data checking and visualization. Demographic and clinical characteristics (including but not limited to age, gender, PTSD, BDI-II scores, BAI scores, and the Life Experiences Survey for the six-month and one-year outcomes) will be compared between groups using t-tests, chi-square tests, Wilcoxon rank-sum tests or Fisher’s exact tests as appropriate to check randomization. Any variable that is found to be different between the groups at the p<0.10 level, and is a plausible potential confounder in consideration of the association under investigation, will be included as a covariate in the analyses described below. This includes an examination as to whether suicide risk variables will be included as covariates.

Given that the aim of the RCT is to test the efficacy of SHUTi, we will perform a per-protocol analysis such that those participants who do not have post-intervention data will not be included in the analysis. Additionally, we will perform a sensitivity analysis using the last value carried forward for those who drop out. This conservative contrast will give some insight into the effectiveness of the intervention and bolster the efficacy findings if they are consistent with the primary analysis.

Primary Aim, Hypothesis 1.1. The primary analyses will be the comparison between groups of the change in 1) insomnia symptom severity as measured by the ISI, 2) physical health as measured by the physical component of the Veteran’s SF-36, and 3) mental health as measured by the mental component summary score on the Veteran’s SF-36, from Time 1 to Time 2. Statistical inference regarding the difference between intervention groups will be based on the estimated coefficient for a group indicator variable in each of three analysis of covariance models with the change from Time 1 to Time 2 for each outcome serving as the dependent variable. Additional covariates will include the baseline value of the outcome to improve precision of the estimate, and any potential confounders discovered in the randomization check. To control for the three primary comparisons, the significance level will be set to 0.0167.

Hypothesis 1.2 and 1.3. A similar analysis will be performed for the above noted outcomes on the change from Time 1 to Time 3 and to Time 4 to determine persistence of group differences to six months and one year post intervention. Additionally, a sensitivity analysis will be performed utilizing a mixed-effects model and all available data, with estimation of the change from Time 1 to Times 2, 3 and 4. The sensitivity analysis will be evaluated for each hypothesis and if it is not consistent than the results for the specific hypothesis will be considered inconclusive.

Exploratory Objective 1.4. For the exploratory outcomes of sleep diary variables (sleep efficiency, sleep onset latency, and wake after sleep onset), risk for SDV (ASIQ), anxiety (BAI), depression (BDI-II), PTSD symptoms (PCL-5), the methods outlined above for the primary analyses will be utilized for the change from Time 1 to Time 2, Time 3, and Time 4.

**G. Summarize Knowledge to be Gained:**

The current study aims to determine the efficacy of SHUTi within the OEF/OIF/OND cohort by comparing the effects of SHUTi to those of an educational website control. In addition to providing data on whether this innovative intervention could be a frontline EBT for Veterans, the proposed RCT will provide critical information on ways to tailor and improve the intervention for future implementation needs. SHUTi is an innovative approach to overcome treatment access barriers, especially for Veterans not eligible for VA care. Furthermore, both short- and long-term follow-up data will provide preliminary evidence as to whether SHUTi produces gains in other comorbid conditions and symptoms in a Veteran population.
