# Supplementary material for: Efficacy of an Internet-Delivered Intervention for Improving Insomnia Severity and Functioning in Veterans: Randomized Controlled Trial
Source: JMIR Ment Health. 2023 Nov 24;10:e50516. doi: 10.2196/50516 (PMC10709797; doi:10.2196/50516)
Supplement: Multimedia Appendix 2 [file mental_v10i1e50516_app2.docx]

**Exploratory Measures**

The *Adult Suicidal Ideation Questionnaire* (ASIQ; Reynolds [47], 1991) has demonstrated high test-retest reliability and internal consistency in clinical and nonclinical samples (Batterham et al [84], 2015), including the current sample (α’s ranged from 0.96-0.97 across time points). The *Beck Anxiety Inventory* (BAI; Beck et al [48], 1988) demonstrated high internal consistency (α’s ranging from 0.94-0.95). Each item is rated on a 4-point Likert scale, with higher numbers suggesting greater degrees of anxiety. The *Beck Depression Inventory II* (BDI-II; Beck et al [82], 2011) demonstrated high internal consistency (α’s of 0.94-0.96). *Posttraumatic Stress Disorder Checklist for DSM-5* (PCL-5; Weathers, et al [83], 2012) scores can be used to create a provisional PTSD diagnosis (symptoms endorsed at “moderately” or higher in congruence with DSM-5 diagnostic rules) or probable PTSD diagnosis (PCL-5 total score ≥33; Marx et al [85], 2022). The PCL-5 demonstrated high internal consistency in the current sample (α range 0.96-0.97). Each item is scored on a 5-point scale, and total scores range from 0-80.

**Results**

At T1, participants on average endorsed moderate depression severity on the BDI-II and mild anxiety severity on the BAI. Approximately half of the sample endorsed symptoms on the PCL-5 indicative of provisional or probable PTSD. On average, most participants endorsed relatively low frequency of past-month suicidal thoughts on the ASIQ.
